# Patterns of biomarker expression in patients treated with primary endocrine therapy – a unique insight using core needle biopsy tissue microarray

**DOI:** 10.1007/s10549-020-06023-4

**Published:** 2020-11-23

**Authors:** R. M. Parks, M. A. Albanghali, B. M. Syed, A. R. Green, I. O. Ellis, K-L. Cheung

**Affiliations:** 1 Nottingham Breast Cancer Research Centre, University of Nottingham, Nottingham, UK; 2 Public Health Department, Faculty of Applied Medical Sciences, Albaha University, Al Bahah, Saudi Arabia; 3 Medical Research Centre, Liaquat University of Medical and Health Sciences, Jamshoro, Pakistan; 4 School of Medicine, Royal Derby Hospital Centre, University of Nottingham, Uttoxeter Road, Derby, DE22 3DT UK

**Keywords:** Core needle biopsy, Tissue microarray, Primary breast cancer, Older women, Primary endocrine therapy

## Abstract

**Purpose:**

Prediction of response to primary endocrine therapy (PET) in older women is based on measurement of oestrogen receptor (ER), progesterone receptor (PgR) and human epidermal growth factor (HER)-2. This study uses a unique method for construction of core needle biopsy (CNB) tissue microarray (TMA), to correlate expression of a panel of 17 biomarkers with clinical outcome, in patients receiving PET.

**Methods:**

Over 37 years (1973–2010), 1758 older (≥ 70 years) women with operable primary breast cancer were managed in a single institution. Of these, 693 had sufficient good-quality CNB to construct TMA, of which 334 had ER-positive tumours treated by PET with a minimum of 6-month follow-up. A panel of biomarkers was measured by immunohistochemistry (ER, PgR, HER2, Ki-67, p53, CK5/6, CK 7/8, EGFR, BCL-2, MUC1, VEGF, LKB1, BRCA1, HER3, HER4, PTEN and AIB1). Expression of each biomarker was dichotomised into ‘low’ or ‘high’ based on breast cancer-specific survival (BCSS).

**Results:**

From the panel of biomarkers, multivariate analysis showed:

- High ER (*p* = 0.003) and PgR (*p* = 0.002) were associated with clinical benefit of PET at 6 months, as opposed to progressive disease.
- High ER (*p* = 0.0023), PgR (*p* < 0.001) and BCL-2 (*p* = 0.043) and low LKB1 (*p* = 0.022) were associated with longer time to progression.
- High PgR (*p* < 0.001) and low MUC1 (*p* = 0.021) were associated with better BCSS.

Expression of other biomarkers did not show any significant correlation.

**Conclusions:**

In addition to ER and PgR; MUC1, BCL-2 and LKB1 are important in determining the outcome of PET in this cohort.

**Electronic supplementary material:**

The online version of this article (10.1007/s10549-020-06023-4) contains supplementary material, which is available to authorised users.

## Introduction

Surgery has better locoregional control of primary breast cancer compared to primary endocrine therapy (PET) in older women [1]. The 2012 joint guidance from the International Society of Geriatric Oncology and European Society of Breast Cancer Specialists [2] recommend that PET should only be offered to patients with oestrogen receptor (ER)-positive tumours with a life expectancy of 2–3 years despite optimisation of medical conditions. Despite this, around 40% of older women with primary breast cancer in the UK are treated by PET [3–5]. Determining who should have PET can be quite subjective and dependent on interpretation of frailty and impact of comorbidity on quality of life. Patients report good satisfaction and low treatment morbidity with PET [2]. Furthermore, surgery and PET have been shown to have similar survival outcomes for up to 5 years [6]; thus, PET appears to be an attractive treatment option in some patients.

Overall, greater than 30% of patients with ER-positive primary breast cancer treated by surgery relapse despite endocrine therapy [7–9]. In addition to this, initiation of PET can take weeks to months before any clinical benefit is seen, and there is an unfortunate group of patients who exhibit no response following this.

Currently prediction of response to PET can only be based on the routinely measured receptors on breast cancer biopsy samples, i.e. ER, progesterone receptor (PgR) and human epidermal growth factor receptor 2 (HER2).

Expression of ER is heterogeneous and predictive value limited, as only 50–70% of patients with ER-positive tumours respond clinically to neoadjuvant endocrine therapy [10–12]. Progesterone expression is associated with good prognosis in ER-positive breast cancer, although its predictive role in endocrine therapy response remains unclear. In the adjuvant setting, PgR expression is associated with greater benefit from endocrine therapy in some studies [13, 14], but not universally [15]. Whilst measurement of HER2 is used for prediction of anti-HER2-targeted therapy, it is also generally associated with a poor response to endocrine therapy [16, 17].

This variability indicates the need for other biomarkers that can predict those likely to respond to treatment and help to determine prognosis, specifically in the setting of primary therapy. Overall research in this area is limited. This is mainly due to the restriction of limited volume of tissue available for research from diagnostic core needle biopsy (CNB). On the other hand, with surgically treated patients, there is less of an issue regarding tumour tissue availability as surgical excision and conventional tissue microarray (TMA) technique can amplify tissue to meet the research requirements.

Following the development of a technique in our group to construct TMAs from diagnostic CNB, irrespective of primary treatment, we have successfully measured a large panel of biomarkers in these CNB TMAs [18].

The goal of this present study was to investigate, using CNB TMAs, the patterns of biomarker expression in patients treated by PET and their relationship to clinical outcome.

This was achieved through measurement of a panel of biomarkers and correlation with (i) clinical response to PET; (ii) time to progression (TTP) of disease and (iii) breast cancer-specific survival (BCSS).

## Methods

### Patient group

Over a 37-year period (1973–2010), 1758 older (≥ 70 years) women with early operable (< 5 cm, T0-2, N0-1, M0) primary breast cancer were managed in a dedicated clinic in Nottingham. Clinical information was available from diagnosis of breast cancer until death or last documented follow-up and has been described previously [4, 19].

### Selection of patients for the current study

It was possible to obtain diagnostic CNB blocks from 1221 of the overall cohort. Of these, 693 cases had blocks with sufficient tumour tissue to construct CNB TMAs. From this group, 639 had tumours which were ER-positive. Positivity was defined as a histochemical (H) score ≥ 1 and measured positive on both diagnostic CNB and CNB TMA in 457 cases, diagnostic CNB alone in 163 cases (measurement on CNB TMA inconclusive) and CNB TMA alone in 19 cases (diagnostic CNB result not available). Out of the ER-positive cases, 386 patients received PET and 334 had a minimum of 6-month follow-up data. Tamoxifen was the commonest endocrine agent used (67.4%), followed by anastrozole (18.9%); the remaining patients had combined therapy. This information has been summarised in Supplementary File 1.

### Construction of CNB TMA

Construction of TMAs from CNB was performed using a novel technique developed in our group [20, 21]. Briefly, based on tumour tissue availability, haematoxylin and eosin (H&E) slides and paraffin CNB blocks were reviewed and multiple areas of 4 mm of tumour were marked from each case, retrieved with skin biopsy punch. Taking into account the estimated average of continuous tumour cell (≥ 3 cm) available from diagnostic biopsy, the vertical re-arrangement method of transferring biopsy was chosen to be used and implanted in Agarose-paraffin TMA blocks. An Agarose-paraffin block with a capacity of 54 core/block was used.

### Measurement of panel of biomarkers

Immunohistochemical (IHC) staining of 17 biomarkers was performed using StreptAvidin Biotin Complex and EnVision methods (DakoCytomation) [22]. The biomarkers measured were ER, PgR, Ki-67, epidermal growth factor receptor (EGFR), HER2, HER3, HER4, p53, cytokeratins CK5/6 and CK7/8, Mucin (MUC)1, liver kinase B1 (LKB1), breast cancer-associated gene (BRCA) 1, B-cell lymphoma (BCL)-2, phosphate and tensin homologue (PTEN), vascular endothelial growth factor (VEGF) and amplified in breast cancer 1 (AIB1).

The expression of biomarkers was assessed using the H-score scoring system (range 0–300) [23] with the exception of HER2, which was scored using the Herceptest scoring system [24] and involves scoring the staining of the membrane (0–3). A TMA core with < 15% tumour material was deemed inadequate and not scored.

Given that we used a historical CNB samples and a novel technique to construct TMA from CNB (with a different diameter in terms of section face comparing to standard technique), we hypothesised that these two factors may affect the antigenicity of targeted markers. Therefore, to estimate the prognostic value of the biomarkers, expression of each biomarker was dichotomised using X-tile software (Version 3.6.1, 2003–2005, University of Yale, Yale, USA [25]) into ‘low expression’ and ‘high expression’, based on observed differences in survival over time.

### Correlation of biomarker expression with clinical response to PET

Assessment of response to PET was done using the Union for International Cancer Control (UICC) criteria [26]. Clinical benefit (CB) was defined as complete or partial response, or stable disease at 6 months. Biomarker expression (either ‘high’ or ‘low’) was correlated with CB.

### Correlation of biomarker expression with time to progression (TTP)

Biomarker expression (either ‘high’ or ‘low’) was correlated with TTP, which was defined as time from diagnosis to progressive disease (progression of primary tumour or metastatic spread, as defined by UICC criteria).

### Correlation with BCSS

Biomarker expression (either ‘high’ or ‘low’) was correlated with BCSS, which was calculated from date of diagnosis to death from breast cancer.

### Statistical analysis

The statistical package SPSS was used for data collection and analysis. Cox-regression model was used to quantify the effect of a biological variable on a clinical variable. A 2-tailed *p*-value of less than 0.05 was determined to be statistically significant.

Results were reported as per Reporting Recommendations for Tumour Marker Prognostic Studies (REMARK) criteria [27].

## Results

### Cohort demographics

The characteristics of the cohort are described in Table 1. The median age of patients was 81 years (range 70–99). Most patients had a grade 2 tumour measuring ≤ 3 cm, with moderate expression of ER. Multivariate analysis of standard histological variables is presented in Supplementary File 2.

**Table 1:** Patient and tumour characteristics of older women with primary breast cancer treated with primary endocrine therapy

Character	Number of patients (percentage)
*Age (years)* Median 82 (range 70–99) 70–79 ≥ 80	*N* = *386* 147 (38) 239 (62)
*Clinical size of tumour (cm)* Median 3 (range 0–5) ≤ 2 2–5 ≥ 5	*N* = *208* 68 (33) 140 (67) 0
*Grade* 1 2 3	*N* = *208* 39 (19) 144 (69) 25 (12)
*ER H-score* Median 158 (range 5–300) 1–50 50–100 101–200 201–300	*N* = *386* 30 (8) 63 (16) 172 (45) 121 (31)
*Survival time* Overall median survival time is 62 months (95% CI 53–71) 5 year overall survival 5 year breast-cancer-specific survival	*N* = *386* 50% 85%
*Cause of death* Death from breast cancer Death from other causes Unknown cause of death	*N* = *210* 43 (20.5%) 146 (69.5%) 21 (10%)

### Measurement of panel of biomarkers

Within a TMA specimen, not all the samples were equally robust to allow IHC staining for each individual sample. Table 2 details the total number of samples possible to test for each biomarker as well as the H-score cut-off to differentiate between high and low expression.

**Table 2:** H-score cut-off between high and low expressions of each biomarker

Biomarker	Total number of samples able to test (N)	Cut-off between high and low expression (H-score*)
ER	334	115
PgR	299	130
Ki-67	299	15
EGFR	300	1
HER2	286	3 +
HER3	308	60
HER4	301	7
p53	298	150
CK5/6	320	1
CK7/8	319	270
MUC1	302	190
LKB1	287	75
BRCA1	255	140
BCL-2	301	265
PTEN	297	170
VEGF	282	80
AIB1	289	18

*Given as H-score for all biomarkers with exception to HER2, for which Herceptest is used

At 6 months after starting PET, 97.3% of patients showed evidence of clinical benefit (Table 3).

**Table 3:** Summary of response to primary endocrine therapy at 6 months based on international union against cancer criteria

Clinical outcome	Number of patients (percentage)
Responses to primary endocrine therapy at 6 months (*N* = 334)	
Complete response (CR)	39 (11.7)
Partial response (PR)	124 (37)
Stable disease (SD)	162 (48.5)
Progressive disease (PD)	9 (2.7)

From the extensive list of biomarkers (Table 4), logistic regression indicated that high expressions of ER (*p* = 0.003) and PgR (*p* = 0.002), compared to low expression, were significantly associated with benefit from PET at 6 months.

**Table 4:** Summary of association between biomarker expression and response to PET at 6 months

Biomarker	Expression status	Response at 6 months N (%)		p-value
		Progressive disease (PD) (*N* = 9, 2.69%)	Clinical benefit (CB) (*N* = 325, 97.3%)	
ER	Low High	7 (7%) 2 (1%)	96 (93%) 229 (99%)	0.001
PgR	Low High	7 (5%) 1 (1%)	132 (85%) 159 (99%)	0.018
Ki-67	Low High	4 (3%) 5 (3%)	115 (97%) 175 (97%)	0.773
EGFR	Low High	9 (3%) 0 (0%)	288 (97%) 3 (100%)	–
HER2	Low High	7 (2%) 1 (100%)	278 (98%) 0 (0%)	–
HER3	Low High	8 (3%) 0 (0%)	252 (97%) 48 (100%)	–
HER4	Low High	6 (3%) 3 (3%)	194 (97%) 98 (97%)	0.989
p53	Low High	6 (2%) 1 (3%)	253 (98%) 38 (97%)	0.924
CK5/6	Low High	7 (3%) 2 (3%)	249 (97%) 62 (97%)	0.866
CK7/8	Low High	8 (3%) 1 (1%)	240 (97%) 70 (99%)	0.428
MUC1	Low High	6 (3%) 2 (3%)	231 (97%) 63 (97%)	0.809
LKB1	Low High	2 (3%) 7 (3%)	61 (97%) 217 (97%)	0.984
BRCA1	Low High	1 (2%) 8 (4%)	50 (98%) 196 (96%)	0.506
BCL-2	Low High	7 (3%) 2 (3%)	214 (97%) 78 (97%)	0.764
PTEN	Low High	8 (3%) 1 (2%)	233 (97%) 55 (98%)	0.553
VEGF	Low High	7 93%) 1 (1%)	208 (97%) 66 (99%)	0.459
AIB1	Low High	2 (6%) 7 (3%)	31 (94%) 249 (97%)	0.313

### Correlation of biomarker expression with TTP

From the cohort of 334 patients, 46% (*n* = 143) showed progressive disease at last follow-up, with a median TTP of 50 months (range of 3–132 months).

**Fig. 1:**
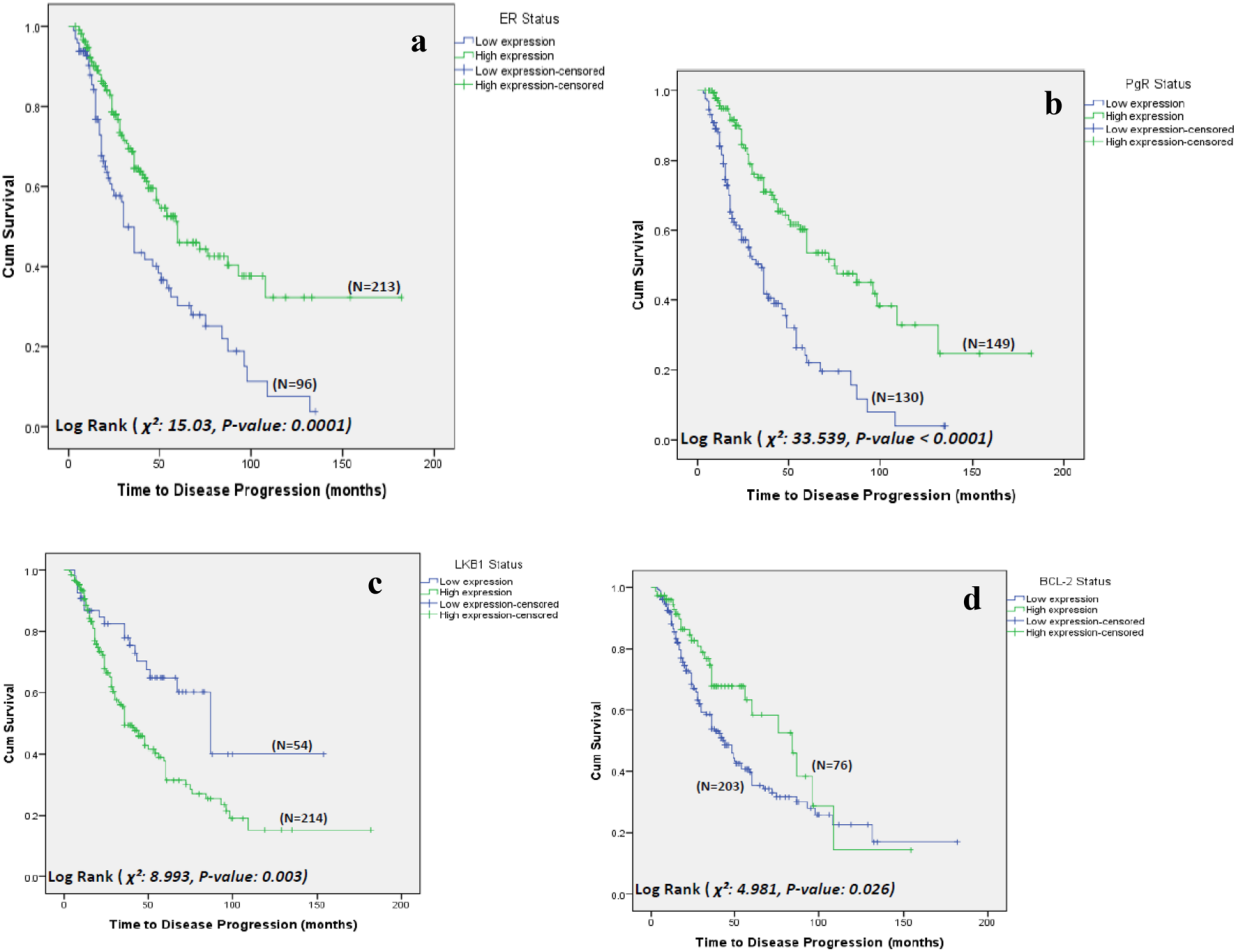
Time to disease progression, older women with primary breast cancer treated with primary endocrine therapy (results of univariate analysis) – stratified based on **a** ER expression, **b** PgR expression, **c** LKB1 expression, **d** BCL-2 expression (*p* values reported are based on univariate analysis)

From the panel of biomarkers tested, multivariate analysis showed that high expression of ER (*p* = 0.023) (Fig. 1a), PgR (*p* < 0.0010) (Fig. 1b), BCL-2 (*p* = 0.043) (Fig. 1c) and low expression of LKB1 (*p* = 0.022) (Fig. 1d) were significant predictors for longer TTP in this cohort.

### Correlation of biomarker expression with BCSS

**Fig. 2:**
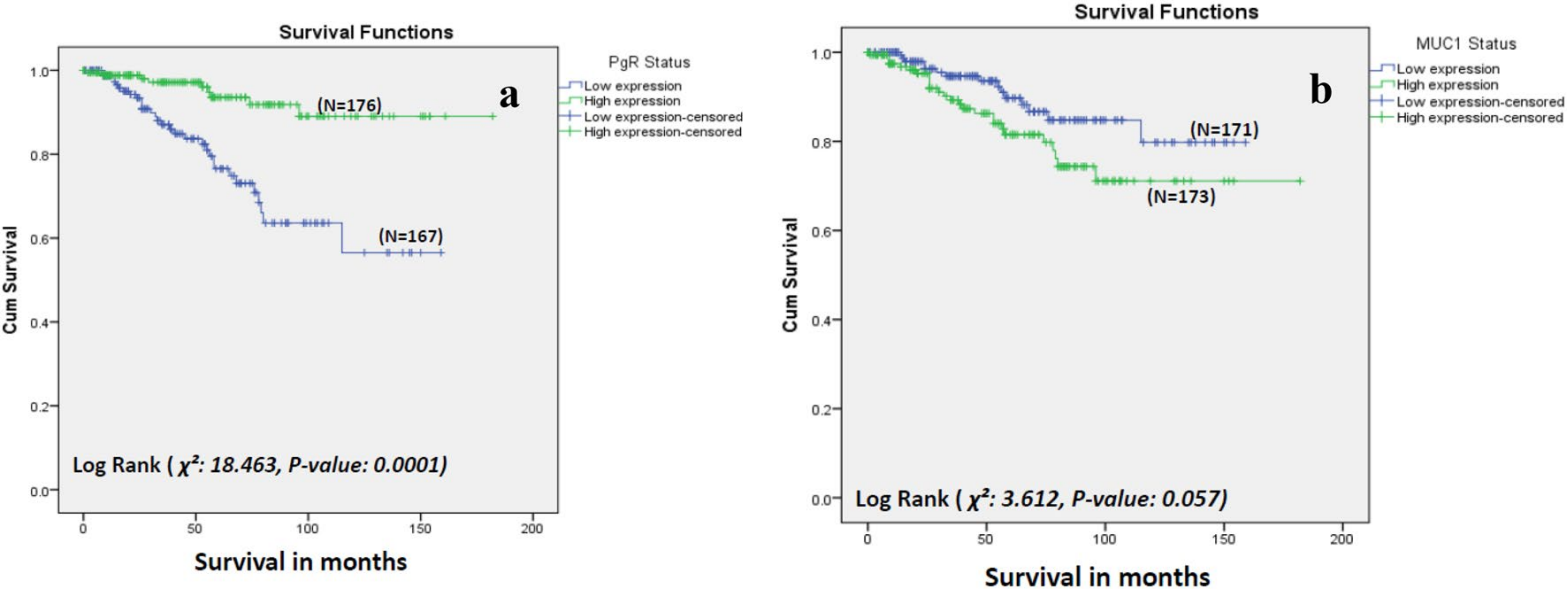
Breast-cancer-specific survival of older women with primary breast cancer treated with primary endocrine therapy (results of univariate analysis) – stratified based on **a** PgR degree of expression and **b** MUC1 degree of expression *(p* values reported are based on univariate analysis)

From the panel of biomarkers tested, multivariate analysis showed that high expression of PgR (*p* < 0.001) (Fig. 2a) and low expression of MUC1 (*p* = 0.021) (Fig. 2b) were significant predictors of increased BCSS in this cohort.

## Discussion

### Correlation of biomarker expression with clinical response to PET

High ER and PgR expressions were associated with complete or partial response to PET at 6 months. These findings add to the growing body of evidence to support PgR as an independent predictive marker of response to endocrine therapy [13, 14, 28]. Tumours that are ER positive are often also PgR positive; thus, the significance of PgR on its own is sometimes overlooked. Expression of PgR serves as an indicator of a functionally intact nuclear ER pathway, so may help to predict which patients will respond to hormone therapies [29]. PgR expression has been associated with greater benefit from endocrine therapy in the adjuvant setting [13, 30].

The present study has clinical implications for the use of PgR as a marker of response to neoadjuvant endocrine therapy. When using endocrine therapy as primary treatment, any CB is important as a marker of survival. When using endocrine therapy in the neoadjuvant setting, achieving a complete or partial response is paramount when downsizing the tumour is the desired outcome.

In our previous study of 536 ER-positive patients within the overall cohort of older women [18], following examination of a panel of biomarkers, cluster analysis was performed and revealed three distinct biological clusters in these cases. Two of the clusters were consistent with well-known subtypes (luminal A like and luminal B like). The third cluster, named ‘low ER luminal’ had higher expression of CK 7/8, BRCA, 2 and BCL-2, and lower expression of ER compared to the conventional clusters.

Patients in the luminal A-like cluster had similar survival outcomes whether they were treated by surgery or PET. Patients in luminal B like had better survival if they had surgery as opposed to PET. Although overall the low ER luminal cluster had lower BCSS compared to both the conventional clusters, within the cluster, patients responded equally well to either surgery or PET. This could be interpreted as both surgery and PET being equally as effective to treat this group of patients and suggests that in addition to conventional features, expression of CK7/8, BRCA2 and BCL-2 may be significant in the role of response to PET.

### Correlation of biomarker expression with TTP

High ER, PgR and BCL-2 and low expression of LKB1 were associated with longer TTP. This is clinically relevant for older frail women who may have competing causes of death; knowing the length of time a cancer can be controlled by PET may impact their treatment decision making.

The protein BCL-2 is involved in the regulation of cell death through interference with the apoptosis pathway and is a ‘pro-survival’ protein in many types of cancer [31]. Biological characterisation of over 14,000 primary breast cancers has shown an increase in BCL-2 expression with age [13]. This present study adds to the evidence that breast cancer in older women has a less aggressive phenotype compared to younger women.

LKB1 is a tumour suppressor gene, of which there is little research in the setting of older women. Studies have shown that LKB1 can inhibit the aromatase enzyme with the AMP-activated protein kinase, supporting its potential role in prediction of response to primary endocrine therapy [32, 33]. In a recent analysis of all patients undergoing surgery within the current cohort of older women described in this present study, older women had higher expression of LKB1 compared to a younger cohort [32]. LKB1 expression was associated with better survival among patients receiving adjuvant endocrine therapy, but TTP was not reported. These results appear contrary to the findings in this present study; low LKB1 expression has been associated with increased TTP of disease.

The authors hypothesise that the contradictory results in these two studies may be related to the context of the studies. In the paper by Syed et al. [32], LKB1 was measured in surgical samples; in the present study, LKB1 has been measured on CNB samples in patients treated by PET. There could be fundamental differences between these two groups of patients due to treatment selection; however, further work is required to confirm this.

### Correlation of biomarker expression with BCSS

High expression of PgR and low expression of MUC1 were associated with better BCSS in this cohort. Correlation with BCSS reflects the biological behaviour of breast cancer in this cohort. Overall survival (OS) in older women is heavily influenced by competing causes of death. Therefore, understanding the biological features which contribute to BCSS and in-depth assessment of frailty, will lead to improved understanding of OS in this cohort.

These findings are consistent with work previously conducted in 536 ER-positive cases of the overall cohort (irrespective of treatment type) [18]. This suggests that outcome is related to the inherent biology of the tumour, regardless of treatment.

Mucin-1 is an epithelial cell surface protein that is overexpressed in up to 90% of breast cancers [34]. MUC1 overexpression has been associated with shorter BCSS in invasive breast cancer but in cases primarily receiving surgical treatment [35, 36] and not specific to older women.

### Summary

In summary, the present study has shown that high expression of ER, PgR and BCL-2 and low expression MUC1 and LKB1 are associated with better outcome in older women treated with PET.

### Strengths of the study

Although the markers found to be significant in this current study have been studied to varying degrees in the literature concerning breast cancer, most research is usually not specific to older women and usually performed in the adjuvant setting. Therefore, this present study is unique in testing a large panel of biomarkers prior to any oncological treatment.

Understanding which biological markers improve BCSS in this cohort in combination with assessment of frailty, may improve OS. This will have impact for consideration of endocrine therapy as neoadjuvant, primary and adjuvant treatment.

### Weaknesses of the study

Due to the small volume of tissue available to construct CNB TMA, it was not possible to construct CNB TMA in all samples and some of the samples degraded on analysis. No direct comparison has been made between SE TMA and CNB TMA to check for concordance.

This study is based in a historic series of patients who were diagnosed with breast cancer as far back as 1973. The main endocrine agent used at the time was tamoxifen, which is not as effective as aromatase inhibitors, which are now routinely used in post-menopausal women. Therefore, survival of this cohort may be not as good overall as expected from a present-day population.

### Future directions

The described technique of CNB TMA construction needs to be validated by other centres. Bioinformatics principles and technologies [37] could help to analyse a large database comparing biomarker expression with clinical outcome, into a format which could be used clinically (e.g. at diagnosis) to help inform patients when making the decision between primary surgery and PET.

## Conclusions

This present study is unique in the examination of a large panel of biomarkers and their application to primary treatment of operable breast cancer in older women. The study adds to the evidence base that breast cancer in older women is generally less aggressive than in their younger counterparts. Alongside the routinely measured markers ER and PgR, BCL-2, MUC1 and LKB1 may be important in determining response to PET and warrant further research.

## Electronic supplementary material

Below is the link to the electronic supplementary material.

Supplementary file1 (PDF 51 kb)

Supplementary file2 (PDF 169 kb)

## Data Availability

This manuscript has no further associated data.

## References

[1] Morgan JL, Reed MW, Wyld L (2014). Primary endocrine therapy as a treatment for older women with operable breast cancer, 2013; a comparison of randomised controlled trial and cohort study findings. Eur J Surg Oncol.

[2] Biganzoli L, Wildiers H, Oakman C, Marotti L, Loibl S, Kunkler I (2012). Management of elderly patients with breast cancer: updated recommendations of the international society of geriatric oncology (SIOG) and European society of breast cancer specialists (EUSOMA). Lancet Oncol.

[3] Ward SE, Richards PD, Morgan JL, Holmes GR, Broggio JW, Collins K (2018). Omission of surgery in older women with early breast cancer has an adverse impact on breast cancer-specific survival. Br J Surg.

[4] Syed BM, Johnston SJ, Wong DWM, Green AR, Winterbottom L, Kennedy H (2012). Long-term (37 years) clinical outcome of older women with early operable primary breast cancer managed in a dedicated clinic. Ann Oncol.

[5] Monypenny I 2003 UK Symptomatic Breast Audit 1.4.2001–31.3.2002. British Association of Surgical Oncology.

[6] Hind D, Wyld L, Beverley C, Reed MW (2006). Surgery versus primary endocrine therapy for operable primary breast cancer in elderly women (70 years plus). Cochrane Database Syst Rev.

[7] Haque MM, Desai KV (2019). Pathways to endocrine therapy resistance in breast cancer. Front Endocrinol.

[8] Piggott L, Silva A, Robinson T, Santiago-Gómez A, Simões BM, Becker M (2018). Acquired resistance of ER-positive breast cancer to endocrine treatment confers an adaptive sensitivity to TRAIL through posttranslational down regulation of c-FLIP. Clin Cancer Res.

[9] Szostakowska M, Trębińska-Stryjewska A, Grzybowska EA, Fabisiewicz A (2019). Resistance to endocrine therapy in breast cancer: molecular mechanisms and future goals. Breast Cancer Res Treat.

[10] Selli C, Dixon JM, Sims AH (2016). Accurate prediction of response to endocrine therapy in breast cancer patients: current and future biomarkers. Breast Cancer Res..

[11] Miller WR, Larionov A, Renshaw L, Anderson TJ, Walker JR, Krause A (2009). Gene expression profiles differentiating between breast cancers clinically responsive or resistant to letrozole. J Clin Oncol.

[12] Colleoni M, Montagna E (2012). Neoadjuvant therapy for ER-positive breast cancers. Ann Oncol.

[13] Daidone MG, Coradini D, Martelli G, Veneroni S (2003). Primary breast cancer in elderly women: biological profile and relation with clinical outcome. Crit Rev Oncol/Hematol.

[14] Nordenskjöld A, Fohlin H, Fornander T, Löfdahl B, Skoog L, Stål O (2016). Progesterone receptor positivity is a predictor of long-term benefit from adjuvant tamoxifen treatment of estrogen receptor positive breast cancer. Breast Cancer Res Treat.

[15] Dowsett M, Cuzick J, Wale C, Howell T, Houghton J, Baum M (2005). Retrospective analysis of time to recurrence in the ATAC trial according to hormone receptor status: an hypothesis-generating study. J Clin Oncol.

[16] Mosly D, Turnbull A, Sims A, Ward C, Langdon S (2018). Predictive markers of endocrine response in breast cancer. World J Exp Med.

[17] Carlomagno C, Perrone F, Gallo C, Laurentiis MD, Lauria R, Morabito A (1996). c-erb B2 overexpression decreases the benefit of adjuvant tamoxifen in early-stage breast cancer without axillary lymph node metastases. J Clin Oncol.

[18] Parks RM, Syed BM, Green AR, Ellis IO, Cheung KL (2020). Biology of oestrogen-receptor positive primary breast cancer in older women with utilisation of core needle biopsy samples and correlation with clinical outcome. Cancers.

[19] Syed BM, Al-Khyatt W, Johnston SJ, Wong DWM, Winterbottom L, Kennedy H (2011). Long-term clinical outcome of oestrogen receptor-positive operable primary breast cancer in older women: a large series from a single centre. Br J Cancer.

[20] Albanghali M, Green A, Rakha E, Aleskandarany M, Nolan C, Ellis I (2015). Construction of tissue microarrays from core needle biopsies – a systematic literature review. Histopathology.

[21] Albanghali M 2016 Biology and clinical outcomes of early primary breast cancer in older women - a study based on core needle biopsy. PhD Thesis, University of Nottingham

[22] Abd El-Rehim DM, Ball G, Pinder SE, Rakha E, Paish C, Robertson JFR (2005). High-throughput protein expression analysis using tissue microarray technology of a large well-characterised series identifies biologically distinct classes of breast cancer confirming recent cDNA expression analyses. Int J Cancer.

[23] Howell A, Harland RNL, Bramwell VHC, Swindell R, Barnes DM, Redford J (1984). Steroid-hormone receptors and survival after first relapse in breast cancer. Lancet.

[24] Jacobs TW, Gown AM, Yaziji H, Barnes MJ, Schnitt SJ (1999). Specificity of herceptest in determining HER-2/neu status of breast cancers using the United States food and drug administration–Approved scoring system. J Clin Oncol.

[25] Camp RL, Dolled-Filhart M, Rimm DL (2004). X-Tile: a new bio-informatics tool for biomarker assessment and outcome-based cut-point optimization. Clin Cancer Res.

[26] Hayward JL, Carbone PP, Heusen JC, Kumaoka S, Segaloff A, Rubens RD (1977). Assessment of response to therapy in advanced breast cancer. Br J Cancer.

[27] McShane LM, Altman DG, Sauerbrei W, Taube SE, Gion M, Clark GM (2006). REporting recommendations for tumor MARKer prognostic studies (REMARK). Breast Cancer Res Treat.

[28] Bardou V-J, Arpino G, Elledge RM, Osborne CK, Clark GM (2003). Progesterone receptor status significantly improves outcome prediction over estrogen receptor status alone for adjuvant endocrine therapy in two large breast cancer databases. J Clin Oncol.

[29] Parks RM JS, Cheung KL 2020 Chapter 7.14 Adjuvant endocrine therapy. Oxford Textbook of General Surgery.

[30] Clark GM, Osborne CK, McGuire WL (1984). Correlations between estrogen receptor, progesterone receptor, and patient characteristics in human breast cancer. J Clin Oncol.

[31] Campbell KJ, Tait SWG (2018). Targeting BCL-2 regulated apoptosis in cancer. Open Biol.

[32] Syed BM, Green AR, Morgan DAL, Ellis IO, Cheung K-L (2019). Liver kinase B1-A potential therapeutic target in hormone-sensitive breast cancer in older women. Cancers.

[33] Azim HA, Kassem L, Treilleux I, Wang Q, El Enein MA, Anis SE (2016). Analysis of PI3K/mTOR pathway biomarkers and their prognostic value in women with hormone receptor-positive, HER2-negative early breast cancer. Transl Oncol.

[34] Stergiou N, Nagel J, Pektor S, Heimes A-S, Jäkel J, Brenner W (2019). Evaluation of a novel monoclonal antibody against tumor-associated MUC1 for diagnosis and prognosis of breast cancer. Int J Med Sci.

[35] Jing X, Liang H, Hao C, Yang X, Cui X (2019). Overexpression of MUC1 predicts poor prognosis in patients with breast cancer. Oncol Rep.

[36] McGuckin MA, Walsh MD, Hohn BG, Ward BG, Wright RG (1995). Prognostic significance of muc1 epithelial mucin expression in breast cancer. Hum Pathol.

[37] Lancashire LJ, Lemetre C, Ball GR (2009). An introduction to artificial neural networks in bioinformatics—application to complex microarray and mass spectrometry datasets in cancer studies. Brief Bioinform.

