# Supplementary material for: Patterns of biomarker expression in patients treated with primary endocrine therapy – a unique insight using core needle biopsy tissue microarray
Source: Breast Cancer Res Treat. 2020 Nov 23;185(3):647–55. doi: 10.1007/s10549-020-06023-4 (PMC7921046; doi:10.1007/s10549-020-06023-4)

Selection of patients for current study from overall cohort of older women with primary breast cancer

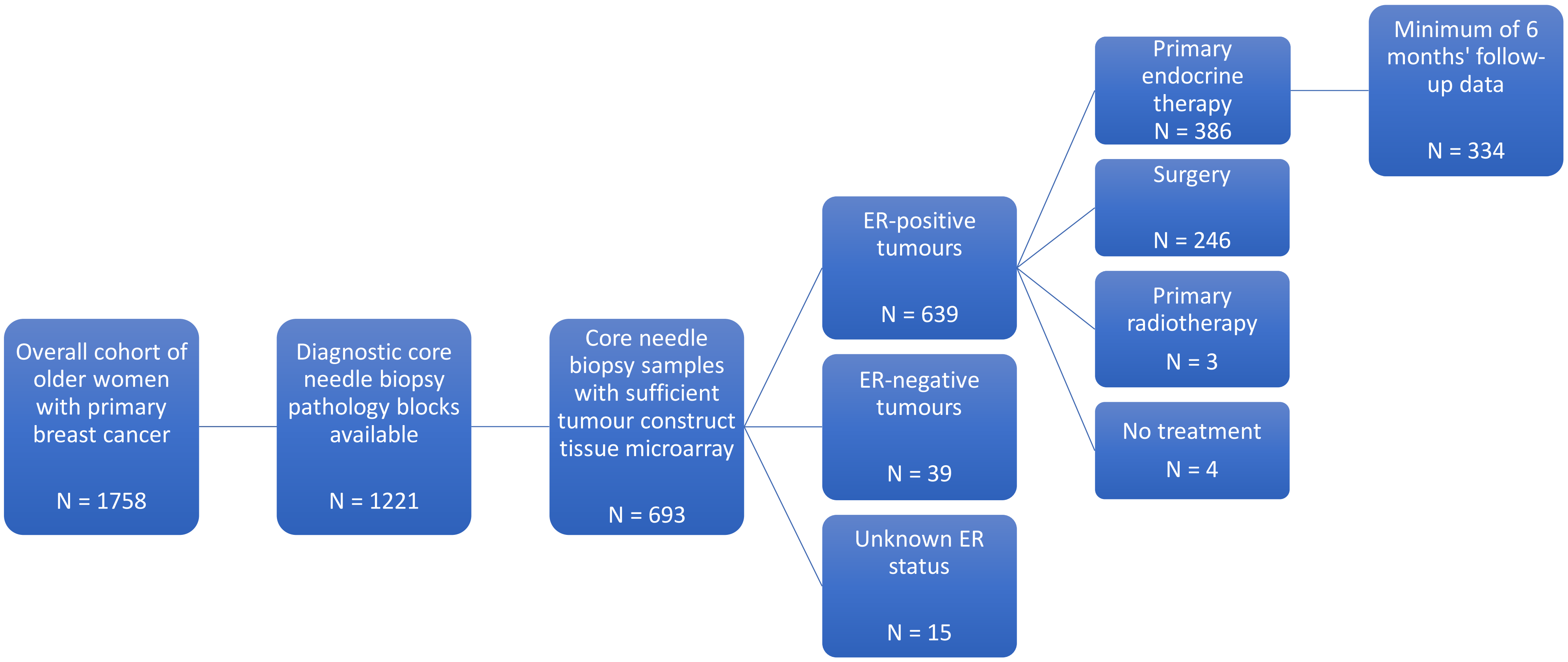

Supplement: Supplementary file 1 — Supplementary file1 (PDF 51 kb) [file 10549_2020_6023_MOESM1_ESM.pdf]
