# Supplementary material for: Patterns of biomarker expression in patients treated with primary endocrine therapy – a unique insight using core needle biopsy tissue microarray
Source: Breast Cancer Res Treat. 2020 Nov 23;185(3):647–55. doi: 10.1007/s10549-020-06023-4 (PMC7921046; doi:10.1007/s10549-020-06023-4)
Supplement: Supplementary file 2 — Supplementary file2 (PDF 169 kb) [file 10549_2020_6023_MOESM2_ESM.pdf]

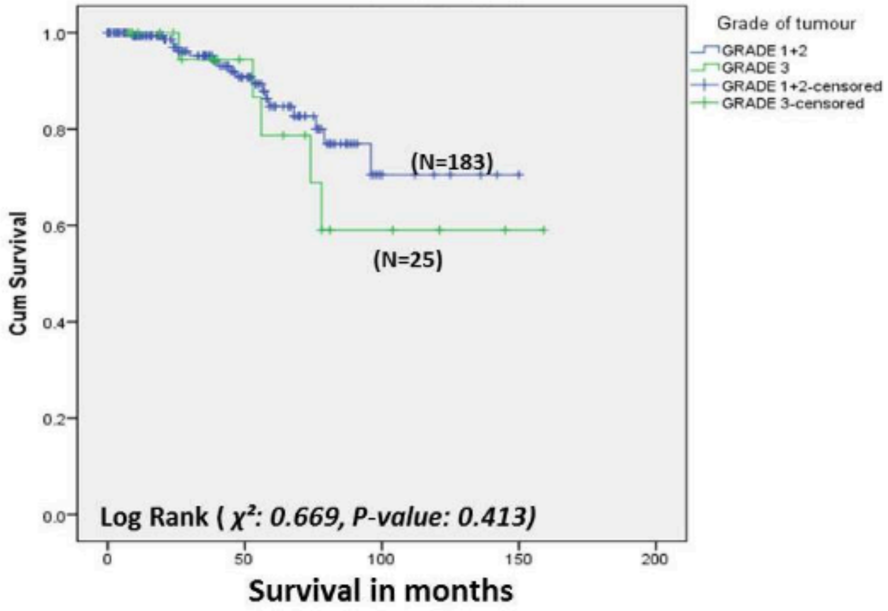

Breast cancer specific survival of older women treated with primary endocrine therapy – stratified curves based on grade of tumour

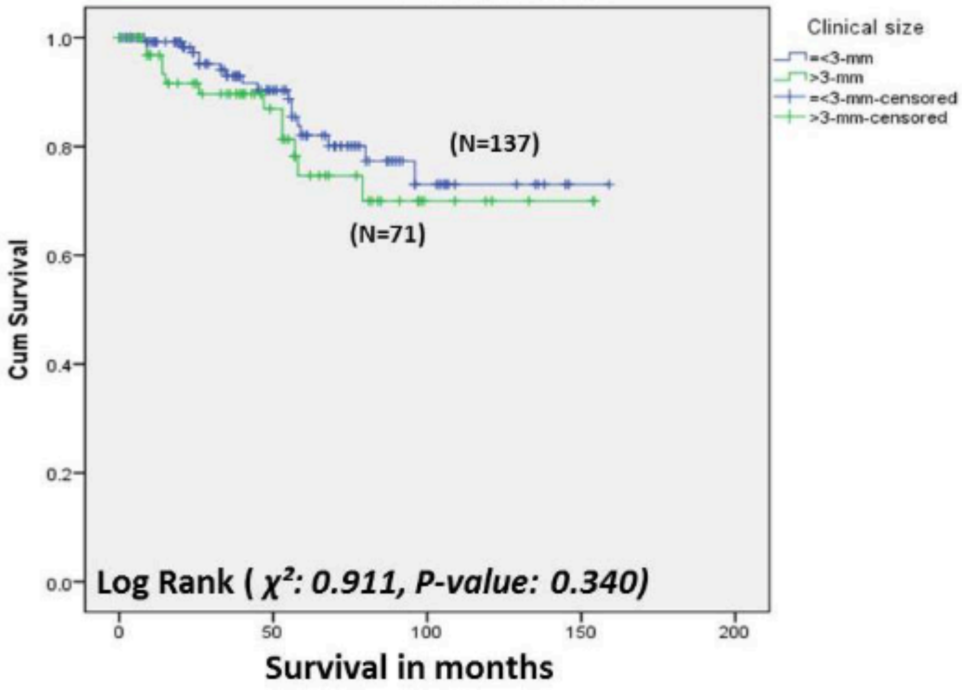

Breast cancer specific survival of older women treated with primary endocrine therapy – stratified curves based on clinical size of tumour
